# Multiscale image analysis reveals structural heterogeneity of the cell microenvironment in homotypic spheroids

**DOI:** 10.1038/srep43693

**Published:** 2017-03-03

**Authors:** Alexander Schmitz, Sabine C. Fischer, Christian Mattheyer, Francesco Pampaloni, Ernst H. K. Stelzer

**Affiliations:** 1 Physical Biology/Physikalische Biologie (IZN, FB 15), Buchmann Institute for Molecular Life Sciences (BMLS), Cluster of Excellence Frankfurt – Macromolecular Complexes (CEF – MC), Goethe Universität – Frankfurt am Main (Campus Riedberg), Max-von-Laue-Straße 15 – D-60348 Frankfurt am Main, Germany

## Abstract

Three-dimensional multicellular aggregates such as spheroids provide reliable *in vitro* substitutes for tissues. Quantitative characterization of spheroids at the cellular level is fundamental. We present the first pipeline that provides three-dimensional, high-quality images of intact spheroids at cellular resolution and a comprehensive image analysis that completes traditional image segmentation by algorithms from other fields. The pipeline combines light sheet-based fluorescence microscopy of optically cleared spheroids with automated nuclei segmentation (F score: 0.88) and concepts from graph analysis and computational topology. Incorporating cell graphs and alpha shapes provided more than 30 features of individual nuclei, the cellular neighborhood and the spheroid morphology. The application of our pipeline to a set of breast carcinoma spheroids revealed two concentric layers of different cell density for more than 30,000 cells. The thickness of the outer cell layer depends on a spheroid’s size and varies between 50% and 75% of its radius. In differently-sized spheroids, we detected patches of different cell densities ranging from 5 × 10^5^ to 1 × 10^6 ^cells/mm^3^. Since cell density affects cell behavior in tissues, structural heterogeneities need to be incorporated into existing models. Our image analysis pipeline provides a multiscale approach to obtain the relevant data for a system-level understanding of tissue architecture.

Three-dimensional *in vitro* cell cultures more closely resemble the cellular microenvironment of cells in tissues than two-dimensional monolayer cultures^1^. Compared to real tissues, they excel with well-defined experimental conditions. Even simple model systems such as monotypic spheroids^2^ or organoids^3^ that show a moderate complexity, provide an adequate and reproducible characterization. Spheroids are three-dimensional multicellular clusters that form through cell aggregation and cell proliferation. With diameters of more than 400–500 μm, they develop a concentric cell layering, in which a necrotic core is surrounded by a layer of quiescent cells and an outer rim of proliferating cells^4^. Many spheroids display properties characteristic of their ancestral tissue such as beating cardiomyocyte spheroids^5^ or aggregates of mouse embryonic stem cells that exhibit axis elongation^6^. Due to their high potential, the applications of spheroids range from fundamental questions underlying cell differentiation and cancer biology to drug discovery and drug response studies^7^.

All these applications depend on the properties of individual cells in a spheroid and all means to retrieve the properties rely on spheroid disintegration or the use of rather small spheroids of less than 200 μm in diameter, which lack the prominent concentric layering and central necrosis. However, morphometric measurements in intact, differently-sized spheroids are needed^8^. Based on histological sections of spheroids, Jagiella *et al*. demonstrated the high potential of retrieving insight into spheroid growth from image-based modelling^9^. Automated image analysis pipelines are required to extend such an analysis to multiple cell lines. Mathematical models of spheroids have shown that changes in the state of each individual cell^10^ must be monitored when studying spheroids. This requires the quantification of the three-dimensional cell environment^11^, since cell properties are affected by cell density^12^, contact inhibition or mechanical pressure^9^,^13^,^14^,^15^.

Phase contrast imaging provides a global but two-dimensional picture of spheroids^16^. Histological sectioning of spheroids allows the quantification of individual cells in a single two-dimensional plane^9^. Confocal and light sheet-based fluorescence microscopy (LSFM) allow the imaging of intact three-dimensional spheroids at the level of individual cells^17^,^18^. For large spheroids, optical clearing methods increase object transparency by achieving refractive uniformity and increase the penetration depth^19^. The combination of optical clearing and LSFM has been used to analyze drug induced cell death in tumor spheroid core regions^20^. Three-dimensional microscopy provides image stacks of fluorescently labelled cell nuclei valuable for the quantitative characterization of the three-dimensional environment in spheroids^21^. Furthermore, nuclei properties correlate with cell proliferation activity^22^ and have shown diagnostic value in oncology^23^.

A large pool of powerful cell nuclei segmentation methods exists, including iterative thresholding^24^, level sets^25^, graph cut^26^, gradient flow tracking^27^, lines-of-sight^28^ or watershed methods^29^. Identifying the approach that is appropriate for a wide variety of datasets, fast and robust with respect to high cell density, as well as variations in cell nuclei volume, shape and dye distribution, has become a major challenge in image analysis. A system-level quantification requires the integration of image segmentation with powerful concepts from other fields such as graph theory, computational topology and spatial statistics. For example, the two-dimensional spatial cell network in breast tissue^30^, malignant glioma^31^ or Hodgkin’s lymphoma^32^ has been characterized by cell graphs. For the implementation of complex image analysis pipelines, software environments such as *Mathematica* (Wolfram Research Inc.) or *Matlab* (MathWorks Inc.) offer comprehensive platforms that integrate well-established image analysis algorithms with a variety of techniques from other computational fields such as graph theory, statistics and computational topology. These platforms can be further extended by integrating packages such as the Insight Segmentation and Registration Toolkit (ITK)^33^, the Visualization Toolkit (VTK)^34^, Fiji^35^ and R^36^.

We developed a robust, multiscale approach for the characterization of large spheroids. Our approach includes three-dimensional cell culture, optical clearing, LSFM imaging and system-level image analysis. Algorithms from graph theory and computational topology complete the segmentation of cell nuclei. The integration of the Laplacian of Gaussian filter into a marker-controlled watershed algorithm provides a robust and accurate cell nuclei segmentation with an F score of 0.88. As a reference, our previous detailed analysis of available tools yielded F scores of at most 0.8^28^. We extended cell graphs to analyze the three-dimensional spatial cell network and introduced the alpha shape as a geometrical model of spheroids. The image analysis pipeline was implemented in *Mathematica* and a user interface is provided.

We applied our image analysis pipeline to characterize size-dependent differences in the internal morphology of spheroids generated from breast cancer cells. Our results revealed the heterogeneity of three-dimensional superstructures that could not have been investigated so far. We detected the concentric cell layering for total cell numbers above 30,000 cells. The relative thickness of the outer region decreases from 75% to 50% of the spheroid radius with increasing cell number. The cell density in spheroids varies between 5 × 10^5^ and 1 × 10^6^ cells/mm^3^. Our image analysis pipeline provides the first quantitative representation of the three-dimensional cell environment in intact, differently-sized spheroids.

## Results

### The combination of optical clearing and LSFM provides insight into the structure of large multicellular spheroids

**Figure 1:**
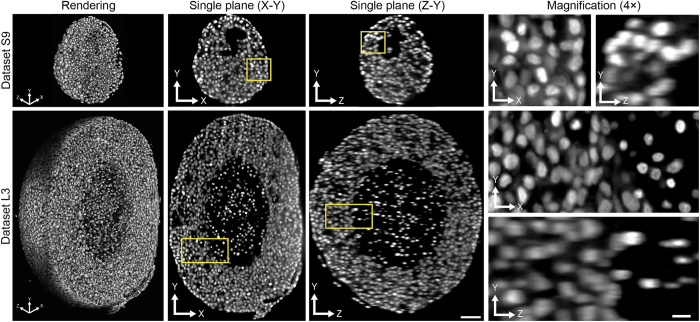
Image quality of three-dimensional datasets. Three-dimensional volume rendering (first column), single planes along X-Y (second column), single planes along Z-Y (third column) and magnification (fourth column) of two spheroids of 500 (upper row, dataset S9) and 10,000 (lower row, dataset L3) seeded cells. For a complete list of datasets see Supplementary Table 4. Renderings in the first column were clipped at about the center of the spheroids and single planes were taken at the same position. Yellow boxes indicate the parts of the images magnified in the fourth column. Microscope: mDSLM. Excitation lens: CZ 5x/NA 0.16. Emission lens: CZ 20x/NA 0.50. Scale bars: 50 μm for the single planes in the second and third column, 10 μm for magnified images in the fourth column.

We applied the complete pipeline to a set of sixteen T47D spheroids that were seeded from 500 to 10,000 cells, developed for two weeks, optically cleared and, finally, imaged *in toto* with LSFM^37^. This resulted in one image stack per dataset with a homogenous signal to noise ratio throughout the entire specimen (Fig. 1). Spheroid diameters range from 150 μm to more than 500 μm.

### Robust and accurate segmentation of cell nuclei in large, densely packed multicellular spheroids

**Figure 2:**
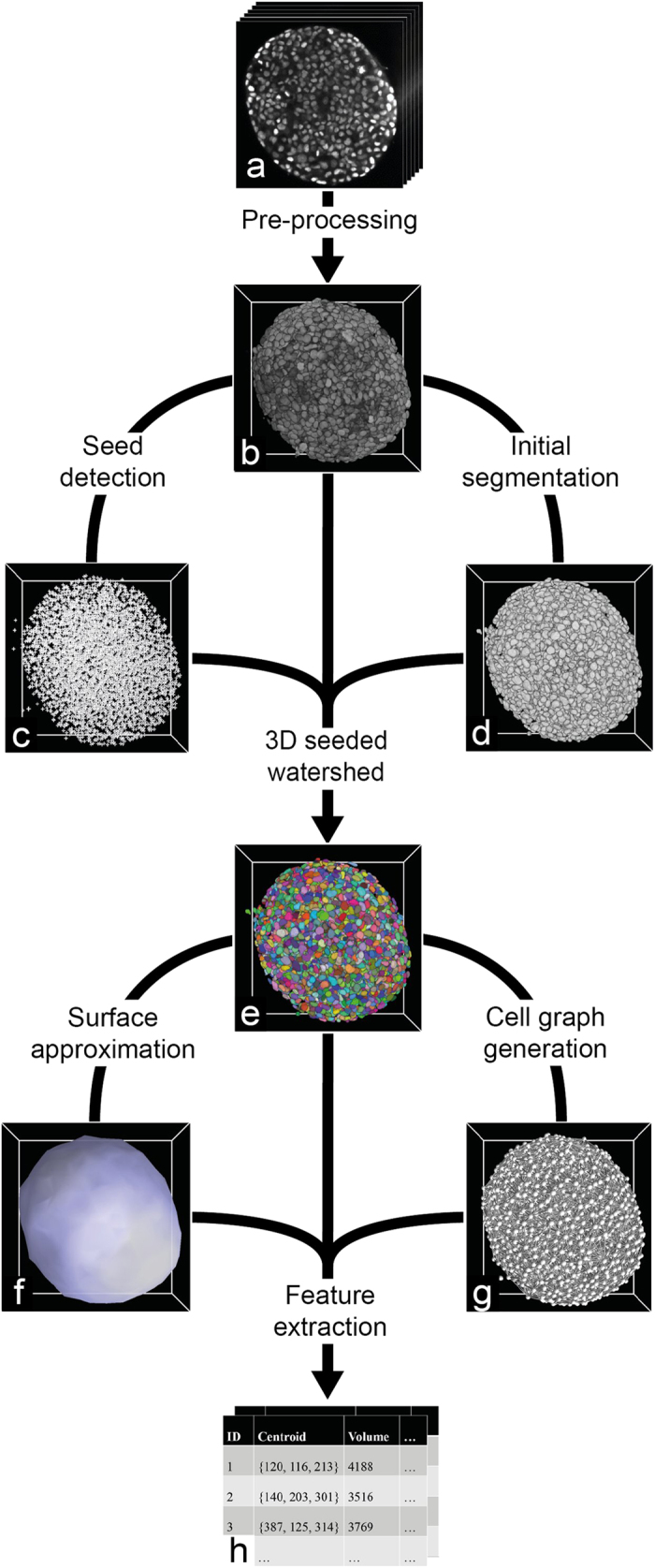
Illustration of the main steps of the automated image analysis pipeline. (**a**) The raw data comprises a stack of two-dimensional optical section images, which in this case consists of 218 planes. (**b**) The image stack is interpolated to obtain isotropic voxels and scaled down by a factor of 0.5. (**c**) Marker positions (indicated as white crosses) are identified by multiscale Laplacian of Gaussian (LoG) filtering of the raw image. (**d**) Regions that contain cell nuclei are separated from the image background by local thresholding of the raw image. (**e**) Identified marker points, the preprocessed raw image and the initial segmentation are subjected to a three-dimensional marker-controlled watershed algorithm, which separates clusters of apparently touching cell nuclei. Different colors indicate individual cell nuclei. (**f**) An alpha shape is constructed from the cell nuclei centroids and the boundary region is extracted as the surface (light blue). (**g**) Cell graphs are generated from vertices, which correspond to the cell nuclei (white spheres), and edges, which indicate the neighborhood relation between two cell nuclei (white lines). (**h**) Features of each individual cell nucleus, the cell neighborhood and the spheroid are extracted and stored in a tabular format.

An accurate cell nuclei segmentation is essential to characterize spheroids quantitatively. However, imaging large samples *in toto* limits the achievable lateral and axial resolution. Furthermore, the high variability of cell nuclei volume and staining in spheroids impedes accurate cell nuclei segmentation. In combination with high cell density, this results in apparently touching cell nuclei in the images that are difficult to separate (Fig. 1, magnification). Our image analysis pipeline identifies cell nuclei accurately and extracts the features required for the characterization of spheroids (Fig. 2).

We use local adaptive thresholding as an initial segmentation of candidate regions. An adaptable three-dimensional, multiscale Laplacian of Gaussian (LoG) filter detects marker points reliably and reproducibly (Supplementary Figure 1). The maximal magnitude of the LoG response is achieved when the scale of the LoG matches the size of the cell nucleus. By incorporating multiple scales, the marker point detection algorithm is more robust towards variations in cell nuclei volume. We measured minimal and maximal radii *r*_*min*_ and *r*_*max*_ of cell nuclei in the datasets (three and six voxels, respectively) to determine the scales *σ*_*min*_ and *σ*_*max*_ of the LoG filter (see Methods section). The resulting marker points are used for a marker-controlled three-dimensional watershed segmentation of the cell nuclei (Supplementary Figure 2). The number of marker points determines the number of objects that are extracted by the watershed algorithm. The processing time for cell nuclei segmentation was less than one hour per dataset.

**Figure 3:**
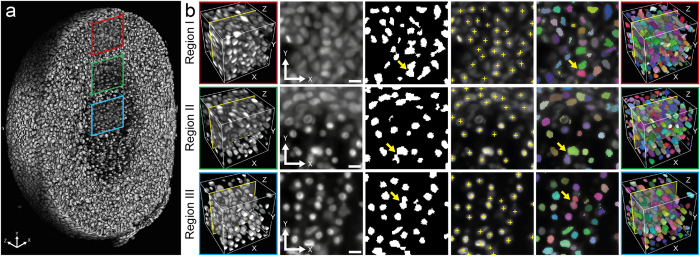
Quality of cell nuclei segmentation in different regions of a large T47D spheroid. (**a**) Three-dimensional rendering of cell nuclei clipped at about the center of an exemplary spheroid (dataset L3, see Supplementary Table 4) that comprises 38,783 cells. Colored boxes indicate three regions that exhibit visually distinguishable properties. Red box: Cell nuclei in region I appear tightly packed. Green box: cell nuclei in region II exhibit diverse morphologies and the intercellular distances differ. Blue box: cell nuclei in region III of the spheroid are small, spherical and appear well separated. (**b**) Exemplary cell nuclei segmentation results for the three regions I, II and III. First column: sub-regions of 100 × 100 × 100 voxels were copied out of the pre-processed raw image. Second column: plane 58 of each sub-region in X-Y view. Third column: the result of the initial segmentation for plane 58. The initial segmentation accurately identifies the foreground in the image. Locations marked with yellow arrows show cell nuclei clusters that are not yet separated. Fourth column: marker points detected by the multiscale LoG filter are overlaid as yellow crosses. Note that for illustration purposes, the marker detection was performed in two dimensions. Fifth column: overlay of the final cell nuclei segmentation of plane 58 after three-dimensional marker-controlled watershed. This step effectively separates apparently touching cell nuclei (yellow arrows). Sixth column: the final segmentation result after three-dimensional marker-controlled watershed of the subregions shown in the first column. Different colors represent individual cell nuclei. Scale bar: 10 μm.

The segmentation performance was evaluated for three different regions I, II and III (Fig. 3a). The regions were chosen to represent the variability in cell nuclei morphology and cellular density (Fig. 3b, second column). In region I, the cell density is high, cell nuclei are morphologically very diverse and apparently touch each other. Region II contains patches of high and low cell density and exhibits a high morphological diversity. Region III contains predominantly small and spherical cell nuclei at a low density. We first evaluated the steps of the segmentation for the three regions qualitatively. The initial segmentation separated image regions that contain cell nuclei from the background. However, this step was not capable of separating clusters of apparently touching cell nuclei (Fig. 3b, third column, yellow arrows).

The marker point detection accurately identified the locations of cell nuclei and is robust in terms of size, shape and intensity variations (Fig. 3b, fourth column). Apparently touching cell nuclei were separated using the marker-controlled watershed algorithm, which was initialized at marker points identified in the marker point detection step. Borders between apparently touching cell nuclei were reconstructed and resulting cell nuclei shapes are now adequately represented (Fig. 3b, fifth column). In summary, cell nuclei in all three tested regions were accurately identified in the final segmentation. To confirm our findings of the visual inspection, we conducted a quantitative performance evaluation of the cell nuclei segmentation (Supplementary Table 1). We generated ground truth datasets (GT) for the three regions (shown in Fig. 3b) by visually extracting the locations of all cell nuclei from dataset L3 (Supplementary Table 4) with a custom program. To assess the segmentation performance, the number of correctly detected (true positives), falsely detected (false positives) and undetected cell nuclei (false negatives) were determined by matching the centroids in the GT with those resulting from the segmentation. We then derived the metrics recall, precision and F score. Out of 252 cell nuclei in the GT in region III, 216 cell nuclei were correctly identified, 36 were not detected and 9 were falsely detected (recall: 0.86, precision: 0.96, F score: 0.91). For region II, 230 out of 269 were correctly identified, 39 were not detected and 32 were falsely detected (recall: 0.86, precision: 0.88, F score: 0.87). In region I, the segmentation performance was 198 true positives out of 233 cell nuclei, 35 false negatives and 24 false positives (recall: 0.85, precision: 0.89, F score: 0.87). Overall, the number of cell nuclei detected by the segmentation is similar to that in the GT in all three regions. In total 110 false negatives and 65 false positives were obtained in all regions, indicating high precision and a low rate of under-segmentation (average recall: 0.86, average precision: 0.91, average F score: 0.88). The increased number of false positive detections in regions II and III indicates that cell nuclei segmentation in these regions is rather difficult. In region III, where cell nuclei have a similar shape and are less densely packed, the performance was slightly better. The obtained F scores indicate high efficiency and robustness of the proposed cell nuclei segmentation. Our previous, detailed performance evaluation of established nuclei segmentation approaches for T47D spheroids^28^ yielded F scores less or equal to 0.8.

The range parameters for the initial segmentation and marker point detection are the main parameters that need to be adjusted for cell nuclei segmentation. The average diameter of the cell nuclei provides a good starting value for these parameters. A user interface allows easy fine-tuning of the parameter values (Supplementary Figures 3–5). For the underlying datasets, the same set of empirically determined parameter values was used (Supplementary Table 2, Supplementary Figure 5). However, we observed a robustness of the segmentation results with respect to changes in the parameter values.

### Quantitative features characterizing spheroids

**Figure 4:**
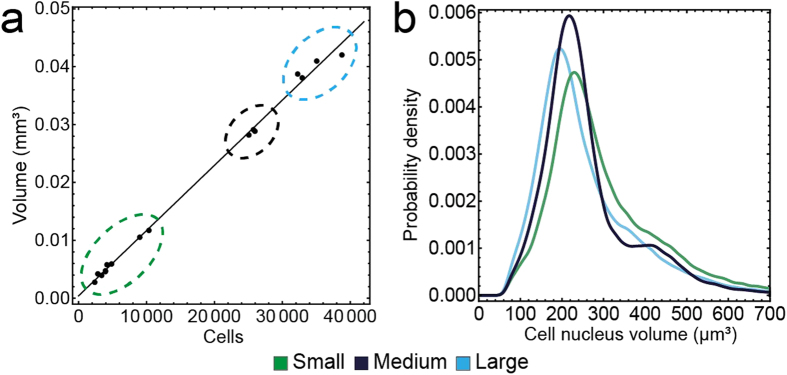
Clustering of datasets results in three groups. (**a**) Plot of spheroid volume versus number of cells detected in the spheroid for all datasets. The measurements are well fitted by a linear model with slope 1,118 μm^3^/cell, Automated clustering of the datasets according to cell number and spheroid volume, returned three groups: small (nine datasets, green ellipse), medium (three datasets, black ellipse) and large spheroids (four datasets, light blue ellipse). (**b**) Smoothed histogram of the cell nuclei volume distribution of small, medium and large spheroids.

We distinguish low level features of the cell nuclei that are directly obtained from the segmentation and higher order features of the cell neighborhood and the whole spheroid. The output of our image analysis pipeline is a comprehensive set of features (Supplementary Table 3). The alpha shape was computed for the cell nuclei centroids and the boundary region was extracted as the spheroid surface (Fig. 4a). A value of 90 voxels for alpha led to a smooth approximation of the surface (Supplementary Figures 6 and 7). From the alpha shape and the extracted surface, we determined the volume, surface area, centroid and the minimal distance of the centroid to the surface.

We observed a strong positive correlation between the number of seeded cells and the spheroid volume (Pearson’s correlation coefficient: 0.95). In addition, the spheroid volume was found to be proportional to the final number of cells and we obtained an increase in volume of 1,127 μm^3^ per cell (Fig. 4a). This indicates a robust linear relation that exists despite the differences in the internal structure of small, medium and large spheroids. Based on automated cluster analysis (partitioning around medoids with squared Euclidean distance) of spheroid volume and cell number, we separated the datasets into three groups of small (n = 9), medium-sized (n = 3) and large (n = 4) spheroids. We determined the mean and standard deviation of the cell numbers (small: 4,977 ± 2,766, medium: 25,582 ± 454, large: 34,742 ± 2,941) and the mean and standard deviation of the spheroid volume (small: 6.0 × 10^6^ ± 3.1 × 10^6^ μm^3^, medium: 2.7 × 10^7^ ± 0.04 × 10^7^ μm^3^, large: 4.0 × 10^7^ ± 0.2 × 10^7^ μm^3^, Supplementary Table 4).

The volumes of the identified cell nuclei were not normally distributed (Fig. 4b). All three distributions exhibit a peak around 250 μm^3^ and an additional shoulder at around 450 μm^3^. Focusing on cell nuclei with volumes between 300 and 600 μm^3^, we did not observe a pattern in their spatial localization (Supplementary Figure 8). All distributions were asymmetric with positive skewness (small: 1.77, medium: 1.71, large: 1.71) and high kurtosis values (small: 8.48, medium: 7.63, large: 7.70). We obtained the median and the median absolute deviation (MAD) values for each spheroid group and found that the median cell nucleus volume in medium and large spheroids was slightly lower compared to small spheroids (small: 267 ± 68 μm^3^, medium: 234 ± 51 μm^3^, large: 225 ± 61 μm^3^). This information does not provide enough detail to analyze differences between the spheroid groups.

Thus, we computed the distance of each cell nucleus to the surface of the spheroid to determine its relative position in the spheroid. For a comparison between different datasets, the location is defined as the normalized shortest distance to the surface (NDS) such that cell nuclei with an NDS of 0 are at the surface and those with an NDS of 1 are in the center of a spheroid. We extended cell graphs to three spatial dimensions and computed two graph representations that capture the spatial arrangement of cells within spheroids. In these graphs, vertices represent the cell nuclei and pairs of vertices are connected by edges representing a pairwise neighborhood relationship. We distinguish between the proximity cell graph (PCG), in which edges are solely created according to the Euclidean distance and the Delaunay cell graph (DCG), in which edges are furthermore only possible between vertices that are connected by a line in the corresponding Delaunay triangulation. For both graphs, we used a distance threshold of 40 voxels for generating the edges. We employed these two graphs to analyze the internal morphology of spheroids. We determined the number of neighbors (i.e., the vertex degree) and the distance to neighbors (i.e., the weights of all incident edges) for each vertex in the graphs as cell density measures. The number of neighbors in the PCG can be interpreted as a measure of local cell density because all cells within a certain distance share the neighborhood relationship. We refer to the number of neighbors for a vertex in the PCG as the local cell density in cells/unit volume (cells/u.v.), where a unit volume corresponds to 65450 μm^3^.

### Local cell density features reveal structural heterogeneity at multiple scales in spheroids

**Figure 5:**
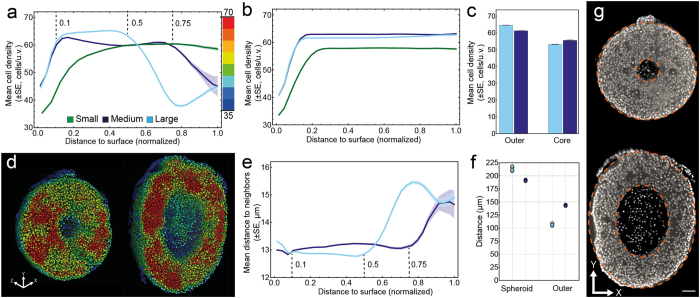
Medium and large spheroids show differences in internal structure. Plot of mean cell density versus the normalized distance to the surface for small, medium and large spheroids (**a**) and the corresponding random cell position (RCP) models (**b**). The RCP models represent an exact analogue of each spheroid with the only difference that cell nuclei are randomly positioned (details are in the Materials and Methods section). The shaded regions indicate the standard error of the mean (SE). Note that in some cases, due to the small error, the shaded region is not visible. Based on the normalized distance to the surface, cell nuclei in the range [0.1, 0.75] for medium spheroids, and [0.1, 0.5] for large spheroids are assigned to the outer region, whereas cell nuclei in the range [0.75, 0.1] for medium and [0.5, 0.1] for large spheroids are assigned to the core region. (**c**) Mean cell density in the outer and core region for medium and large spheroids. (**d**) Three-dimensional rendering of segmented cell nuclei colored according to their corresponding cell density for a medium (dataset M3) and a large spheroid (dataset L2), ranging from blue (35 cells/u.v.) to red (70 cells/u.v.). For a complete list of datasets see Supplementary Table 4. Renderings were clipped at the center of the spheroids. (**e**) Plot of the mean distance to neighbors versus the normalized distance to the surface. The shaded regions indicate the standard error of the mean (SE). Note that in some cases, due to the small error, the shaded region is not visible. (**f**) Radius of medium and large spheroids and thickness of the outer region. (**g**) Raw single planes at the center of the spheroids shown in d with orange dashed lines indicating the boundaries of the outer region. Scale bar: 50 μm.

We inspected all features of the cell nuclei as a function of depth in the spheroid to check for variations along the radial direction. Low level cell nuclei features directly extracted from the segmentation did not show any variation along the radial direction of the spheroids. However, higher order features derived from the cell graphs that characterize local cell density varied along the radial direction in medium and large spheroids (Fig. 5a, Supplementary Figure 9). A prerequisite for any statistical analysis of cell aggregates is that measured features show a significant deviation from randomness^32^,^38^. To assess the randomness of cellular arrangement, we placed cells randomly into the alpha shape of each dataset, computed the cell density features and compared them with those found for the real datasets. Thus, we plotted the mean cell density as a function of depth and compared it to the random cell position (RCP) model^38^ (Fig. 5b). We found that the curves for medium and large spheroids strongly deviate from the RCP model, whereas in small spheroids a similar curve is obtained. Thus, the spatial distribution of cells in medium and large spheroids deviates from randomness, whereas the spatial distribution in small spheroids could be generated by randomly positioning the cells. Consequently, we further analyzed the features capturing local cell density in medium and large spheroids. Cells at the surface have only few neighboring cells (mean cell density ≈ 45 cells/u.v.). At 0.5 NDS, the cell density decreases from 65–40 cells/u.v. in large spheroids, whereas a similar decrease from 60–45 cells/u.v. is observed at 0.75 NDS in medium spheroids (Fig. 5a).

To compare our results to the known concentric cell layering in larger spheroids^4^, we manually subdivided medium and large spheroids into three distinct regions *surface, outer* and *core*. For the separation of surface and outer region we take the transition point between the initial rise and the approximately constant region, resulting in a threshold of 0.1 NDS for medium and large spheroids. For the separation of outer and core region we take the transition point between the approximately constant region and the decreasing part resulting in 0.75 NDS for medium and 0.5 NDS for large spheroids. For the mean cell density, differences were detected for outer and core regions in both spheroid groups (Fig. 5c). In medium spheroids, the mean cell density between outer and core region differs by 9.4% (outer: 61 cells/u.v., core: 56 cells/u.v.), whereas in large spheroids, the difference is 17.7% (outer: 65 cells/u.v., core: 53 cells/u.v.). To cross-check these findings, we colored the segmented cell nuclei according to the cell density (Supplementary Figure 10). Consistent with the quantification, low cell density was observed in the central and surface regions of the spheroids, while the cell density is higher for cells between these two regions (Fig. 5d). Within the identified regions, the cell density was not homogeneous and we observed patches of high cell density. This result also extends to small spheroids (Supplementary Figure 10). To evaluate the contribution of cell divisions to cell density, we manually identified occurring cell divisions in three representative datasets (S9, M2 and L3). We found between one (S9) and five (L3) cells that were at the end of the mitotic phase. For medium and large spheroids, we could not identify any correlation between the measured cell density and the nuclei volume (average Pearson’s correlation coefficient −0.23) or shape (average Pearson’s correlation coefficient −0.11). Similar to the results obtained from the PCG, the mean distance to neighbors determined from the DCG was constant up to 0.75 NDS in medium spheroids and increased to 15.5 μm in the core. In large spheroids, a similar increase was already observed at 0.5 NDS (Fig. 5e). Based on the quantitative measures, we determined the average radius of the spheroids, the average thickness of the outer region and the volume of the outer region (Fig. 5f, Supplementary Figure 11). On average, medium spheroids have a radius of 191.3 μm and large spheroids have a radius of 213.7 μm. As indicated by the previous results, the thickness of the outer region in medium spheroids is larger than in large spheroids (143.5 μm in medium, compared to 106.8 μm in large spheroids) and the volume of the outer region in medium spheroids is smaller than in large spheroids (0.028 mm^3^ in medium and 0.033 mm^3^ in large spheroids, Supplementary Figure 11). We again cross-checked by highlighting the detected border between outer and core region in the raw data (Fig. 5g). We found that the quantitatively identified location of the border fits with the visually observable boundary.

## Discussion

Three-dimensional cell aggregates continue to become more and more important model systems in basic cell as well as in translational biology. Common experiments in three-dimensional cell biology rely on chemical^39^ or mechanical^40^ perturbations of spheroids. Examples include measuring the influence of mechanical confinement on the internal morphology of spheroids or the influence of chemical compounds on cell differentiation, proliferation and viability. These approaches are completed by our method, which enables the quantitative characterization of perturbation effects on spheroids.

Despite recent advances in three-dimensional cell cultures and fluorescence microscopy, any quantitative analysis of spheroids remains complex. We developed a pipeline including three-dimensional cell culture, optical clearing, LSFM imaging and three-dimensional image analysis. Apart from a detailed characterization of spheroids, our image analysis also results in a reduction of large images to simple lists. The number of extracted nuclei features goes well beyond those available in most existing programs^21^,^41^,^42^,^43^,^44^. The output comprises measurements obtained from the segmentation extended by features obtained from graph and topological analysis.

The combination of optical clearing and LSFM resulted in full penetration depth, homogeneous intensity distribution and good axial resolution. We obtained the first comprehensive dataset of high quality images for subsequent quantitative characterization of spheroids at the single cell level. Our image analysis pipeline completes cell nuclei segmentation by powerful methods from graph theory and computational topology.

The cell nuclei segmentation is objective, robust and exceeds the performance of well-established programs such as FarSight and CellProfiler. It is insensitive towards intensity variations and capable of separating apparently touching cell nuclei in regions of different cellular density. The initial local segmentation is less sensitive towards heterogeneous intensity distributions than global thresholding^43^,^44^,^45^. The multiscale LoG approach is capable of reliably detecting marker points for irregular nuclear shapes, varying intensity distributions and in regions of high cell density^42^,^43^,^45^. In conjunction with results from recent studies^43^,^45^, we conclude that the identified combination of methods is a promising candidate for a universally applicable cell nuclei segmentation approach.

We demonstrated that alpha shapes provide a good geometrical model of arbitrarily shaped cell aggregates, based on the positions of cells and a suitable parameter value for alpha. Compared to the commonly used spherical harmonics approach^46^,^47^, the alpha shape excels in applicability (own unpublished results). The computational effort is low and they provide an accurate geometrical model of solid three-dimensional objects of arbitrary shape.

Graph based approaches give a formal and well-researched description of complex, multi-dimensional networks. Cell graphs inherently contain the information to compute global and local features that define the cell aggregate topology and relationships between individual cells^32^. To investigate the local cell structures in the spheroid, we extended cell graphs to three-dimensional spatial networks and implemented two cell graphs: a purely distance-based variant (proximity cell graph, PCG) and a variant that is based on Delaunay triangulation (Delaunay cell graph, DCG). By incorporating the alpha shape surface, these graphs capture structural patterns as a function of depth in spheroids. PCG and DCG model different modes of cell neighborhood. The PCG provides a measure of local cell density and connects all cells that interact across long ranges. Neighborhood in the DCG is an approximation of which cells are in direct contact with each other. For the PCG, the number of edges and hence the computation time increases with the distance threshold. The DCG is less sensitive to the chosen distance threshold, since the number of edges is restricted by the Delaunay triangulation. Since the Delaunay triangulation is the dual form of the Voronoi tessellation, an approximation of cellular shape can be readily obtained from the DCG^44^,^48^. In this study, both cell graphs identified the same structural patterns in spheroids. In the future, features extracted from the segmentation can be incorporated into the cell graphs to derive higher order information of the underlying system, in particular, spatial correlation of cell morphology, cell type or expression profiles.

We applied the complete pipeline to sixteen differently-sized T47D spheroids using identical parameter values for the image analysis. Our pipeline provides quantitative data on cell location for advanced statistical analyses^37^ and cell nuclei volume, cell number and the spheroid diameter for validation and refinement of mathematical models for spheroid growth dynamics^13^. Our approach differs from the classical approaches for studying spheroid growth dynamics. We generated spheroids from fixed numbers of seeded cells and grew them for the same period. This provides the basis for a comparison of the properties of differently-sized spheroids. We found a linear relationship between spheroid volume and cell number despite the differences in cell density distribution between small, medium and large spheroids. Hence, for a clear distinction between spheroids of different internal morphology it is insufficient to analyze such global spheroid properties. By extending the classical measures with results from cell graphs and alpha shapes, we obtained an objective method to detect local structural properties and the boundary between the outer and core region in spheroids. An extensive core region in the breast carcinoma spheroids arises for a total cell number of at least 30,000 cells. Furthermore, we revealed a structural inhomogeneity that goes well beyond the expected concentric layering. The diameter of the core region is not proportional to the diameter of the whole spheroid. In larger spheroids, the outer region is relatively thinner (50% of the spheroid radius) but has a larger volume than in medium-sized spheroids (75% of the spheroid radius). The thickness of the outer region depends on nutrient and oxygen uptake^2^ that vary with spheroid size^49^.

Within the spheroids of all sizes, the cell density varies between 35 and 70 cells/u.v. and patches of higher and lower cell density occur in the outer region. We could not identify any correlation between the cell density differences and cellular growth properties such as cell nuclei volume and the occurrence of cell divisions. The low number of cell divisions matches a cell cycle duration of 72 hours for T47D cells as measured in our laboratory (unpublished). We expect the observed heterogeneity in the cell density to have an impact on individual cells. Studies based on two-dimensional cell cultures have revealed that cell density affects cellular behavior and function^50^ including cell differentiation^51^ and the response to compounds^52^. These findings also extend to real tissues^53^. Hence, the heterogeneity of three-dimensional cell cultures has to be integrated into existing models.

Our approach can be readily applied to analyze spheroid growth dynamics. For example, spheroids could be grown from the same number of seeded cells for different periods to study the development of the internal spheroid morphology as a function of time. This approach could be extended to analyze growth dynamics that exceed the culturing period by incorporating the growth curves for different numbers of seeded cells into one curve. For our data, this approach indicates an exponential growth for the first 28 days, followed by a linear growth (results not shown) in agreement with previous work^13^. Furthermore, these approaches could be extended by antibody staining optimized for spheroids^54^ to link the internal spheroid morphology to cellular properties (e.g. apoptotic, necrotic, proliferative). In particular, previous results have indicated a positive correlation between cell nucleus volume and cell proliferation^22^. We found a uniform distribution of large cell nuclei within spheroids of all sizes (Supplementary Figure 8) that interestingly deviates from the distribution of proliferating cells in recent images of histological sections stained with a proliferation marker^9^. More detailed investigations could shed light on whether this is a cell type specific phenomenon.

**Figure 6:**
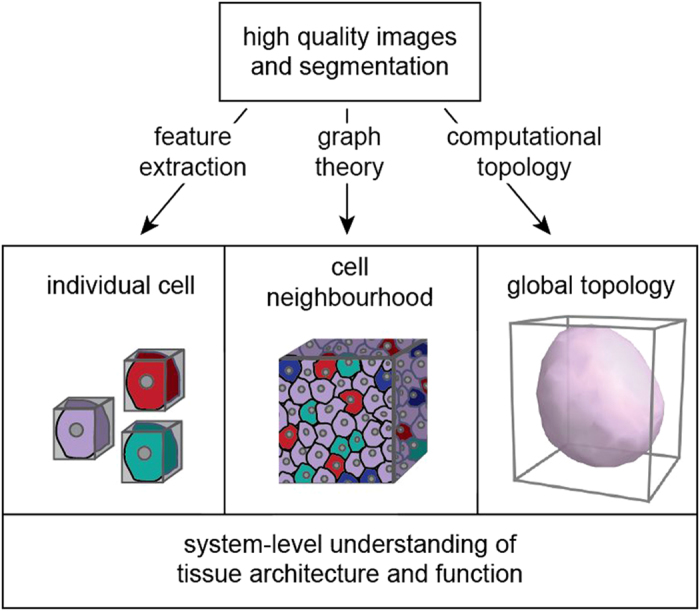
Three-dimensional cell biology requires a multiscale image analysis approach. The established methods for advanced three-dimensional microscopy and image segmentation need to be extended by concepts from other fields including graph theory and computational topology. This will provide a system-level understanding of tissue architecture and function.

Our approach is not restricted to spheroids. Provided high-quality images with homogeneously stained nuclei of approximately convex shape, our image analysis can be applied to characterize any three-dimensional multicellular system at multiple scales, ranging from the single cell level to the cell microenvironment and the whole system (Supplementary Figure 12). The complete pipeline can be extended to specimens of several mm in diameter that would comprise at least several 100,000 cells. We conclude that studies in three-dimensional cell biology need to incorporate quantitative measurements at the level of the single cell, the cell neighborhood and the whole system to draw physiologically relevant conclusions (Fig. 6). A multiscale characterization can only be obtained by integrating powerful algorithms from other computational fields into traditional image analysis approaches.

## Methods

### Spheroid preparation and imaging

#### Spheroid preparation

Spheroids were formed by liquid overlay from non-invasive T47D breast cancer cells. Cell suspensions containing seed numbers of 500, 1,000, 2,000, 5,000 or 10,000 T47D breast carcinoma cells were placed in U-well plates coated with a non-adhesive surface (Thermo Scientific, Nunc HydroCell 96 Well, 174908) that facilitates spheroid formation. The cells were cultured for twelve days in order to obtain mature spheroids. Subsequently, spheroids were chemically fixed, washed, stained for cell nuclei with the fluorescent dye Draq5 and optically cleared with BABB^19^ to minimize light scattering.

#### Image acquisition

Image stacks of optical sections of entire T47D spheroids were acquired with a monolithic Digital Scanned Laser Light Sheet-based Fluorescence Microscope (mDSLM) as described previously^55^. We define the illumination axis as X, the detection axis as Z and the axis orthogonal to X and Z as Y. The excitation light source is a 638 nm laser operated at 1.6 mW and focused with a Carl Zeiss Epiplan-Neofluar 2.5x/NA 0.06 objective lens. A Carl Zeiss N-Achroplan 20x/NA 0.5 objective lens, a 680/60 bandpass filter, a regular tube lens and a high-resolution Andor Neo 5.5 sCMOS camera with a pixel pitch of 6.5 μm are used in the emission path. Image stacks were acquired with an axial pitch of 1.29 μm. The cropped regions of interest result in raw image stack sizes ranging from 230 Megabytes to 2 Gigabytes.

### Cell nuclei segmentation

#### Pre-processing

Isotropic voxels are obtained by rescaling the Z dimension and interpolating missing planes (*ImageZScalingFactor*). This results in isotropic voxels with a pitch of 0.325 μm. For computational efficiency, the resulting three-dimensional image is resized by a factor of 0.5 (*ImageScalingFactor*). In the resized image, the voxels are isotropic with a pitch of 0.65 μm. In the following, the raw three-dimensional image is termed *f*_*r*_(*x, y, z*).

#### Initial cell nuclei segmentation

Noise in *f*_*r*_(*x, y, z*) is reduced by the convolution with a three-dimensional Gaussian kernel of range 3 × 3 × 3 (*NucleiFilterRange*). The average background intensity *t*_*global*_ of the convolved image is determined by Otsu’s global thresholding algorithm^56^. In the initial cell nuclei segmentation step, local thresholding is applied per sectional plane along the dimensions X, Y and Z and the resulting binary images are multiplied to obtain the initial segmentation. For each pixel the local threshold *t*_*local*_ is determined by


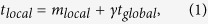


where *m*_*local*_ corresponds to the mean intensity measured in a range of 25 × 25 pixels (*NucleiThresholdRange*) and *t*_*global*_ is the determined background intensity. The factor γ is a parameter of the cell nuclei segmentation that controls the impact of the determined background intensity (*NucleiBackgroundFactor*). For all datasets, γ was set to 0.25. Each pixel with an intensity greater than *t*_*local*_ is set to 1, all others are set to 0. Holes, i.e. regions that are falsely detected as background because of minor segmentation errors, are removed. Therefore, the initial segmentation is inverted to obtain the complement image. Holes are identified as small foreground regions and those smaller than a predefined threshold of 250 voxels are removed. This threshold corresponds to about one quarter of the mean volume of a cell nucleus and was determined prior to segmentation. The image is inverted again to obtain the hole-corrected *f*_*b*_(*x, y, z*).

#### Marker point detection

For the decomposition of connected nuclei in *f*_*b*_(*x, y, z*), we use a three-dimensional marker-controlled immersion watershed algorithm. The marker points are obtained by the multiscale Laplacian of Gaussian (LoG) blob detection algorithm. Thereby, a blob refers to an approximately convex region of a three-dimensional image, in which the intensities vary within a sufficiently small range of values. First, the raw image *f*_*r*_(*x, y, z*) is inverted, resulting in *f*_*i*_(*x, y, z*). The LoG corresponds to first convolving *f*_*i*_(*x, y, z*) by a Gaussian kernel *g*(*x, y, z*; *σ*). Thus, we get


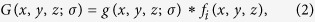


where *σ* is the standard deviation (scale) of the Gaussian kernel *g*. Then the Laplacian operator ∇^2^ is applied to the convolved image to obtain the LoG response. In our approach, the inverted image *f*_*i*_(*x, y, z*) is processed at multiple scales 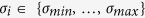. The minimal and maximal scales *σ*_*min*_ and *σ*_*max*_ are determined using the relationship


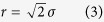


between radius *r* of a blob-like object and the scale *σ* of the LoG. We measured the minimal (*r*_*min*_) and maximal (*r*_*max*_) radius of cell nuclei *a priori* in the images (*NucleiSeedDetectionMinRadius* and *NucleiSeedDetectionMaxRadius*) and used the relationship above to compute *σ*_*min*_ and *σ*_*max*_. To achieve scale-invariance, the LoG response at scale *σ* is normalized by multiplication with *σ*^3^ such that the LoG response at scale *σ* is given by


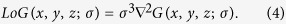


For computational efficiency, we iteratively compute the maximum response 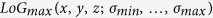 over scales given by


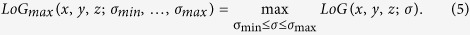


The maximum response 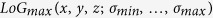 is further processed with a maximum transform for detection of extended maxima. The obtained binary image is multiplied with the initial segmentation *f*_*b*_(*x, y, z*) to discard extended maxima detected in the background. Extended maxima that are in close proximity to each other are merged by increasing the size of the marker points using a morphological dilation operator with a round structuring element of two voxels radius (*NucleiSeedDilation*). The resulting binary image *f*_*m*_(*x, y, z*) specifies the marker points that are used to initialize the subsequent watershed algorithm.

#### Watershed-based decomposition of cell nuclei clusters

We use the immersion watershed algorithm to achieve a decomposition of connected cell nuclei clusters. The algorithm starts at the marker points in *f*_*m*_(*x, y, z*) as sources and iteratively immerses the inverted image *f*_*i*_(*x, y, z*) according to the intensity. Watersheds are built when different sources meet during the immersion process. A partitioning of the image *f*_*i*_(*x, y, z*) into labelled components (1 to number of components) and watersheds (0 s) is obtained. However, the immersion process does not necessarily stop at the border of a cell nucleus. Thus, we multiply the resulting matrix with the initial segmentation *f*_*b*_(*x, y, z*). Objects are selected by incorporating lower and upper volume thresholds (*NucleiMinCount* and *NucleiMaxCount*). Based on the measurements of minimal and maximal radii of cell nuclei, the cell nucleus volume is approximated as a sphere with equivalent radius and we obtained an approximate lower threshold of 250 voxels and an upper threshold of 42,500 voxels. As the final result of the segmentation, we obtain the matrix *w*(*x, y, z*). Each labelled component in *w*(*x, y, z*) represents a cell nucleus found by the segmentation.

#### Evaluation of segmentation performance

We generated three ground truth (GT) datasets by cropping three sub-regions of 100 × 100 × 100 voxels out of the downsized raw images. The centroids of all cell nuclei within the sub-regions were visually identified with a custom program. Based on the generated GT, we determined the number of correctly detected cell nuclei (true positives, TP), the number of cell nuclei that were falsely detected by the segmentation (false positives, FP) and the number of cell nuclei in the GT that were not detected by the segmentation (false negatives, FN). To compute these numbers, we used the following algorithm: if exactly one centroid of the segmentation is found within a spherical neighborhood of twelve voxels of a centroid in the GT, we count it as TP and delete it from the list. If more than one centroid is found within this neighborhood range, the closest one is considered as TP. Based on the number of TP, the FP and FN were obtained using *FP* = *N*_*SC*_−*TP* and *FN* = *N*_*GT*_ − *TP*, where *N*_*SC*_ is the number of centroids determined by the segmentation and *N*_*GT*_ is the number of centroids in the GT. Based on these measurements we derived the metrics recall, precision and F score^28^ with values ranging from 0 (worst performance) to 1 (optimal performance):


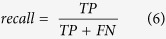



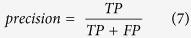



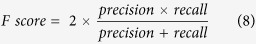


### Feature computation and randomness of the spatial cell distribution

#### Basic cell nuclei features

Based on the segmentation, we extract intensity-related and morphological features of each identified cell nucleus. The morphological features include the volume in number of voxels, centroid, intensity-weighted centroid, mean, minimum and maximum distance to the centroid, number of voxels on the surface and the bounding box. We use principal component analysis to determine the principal directions and the extensions along these directions as a measure of shape and orientation of the cell nucleus. Intensity-related features include the mean, minimum and maximum intensity and the intensity standard deviation. All intensity-related features are rescaled to the interval [0, 1].

#### Surface approximation

We approximate the surface of the spheroid using the alpha shapes approach based on the set *P* of cell nuclei centroids. To discriminate against false positive detections, all points in *P* that are within a distance of 20 voxels are connected (*OutlierDistanceThreshold*). The largest connected component is regarded as the cell aggregate and remaining components are removed. The surface of the cell aggregate is then approximated using Edelsbrunner’s algorithm for alpha shapes^57^ with parameter alpha (*Alpha*). The alpha shape approach is based on a Delaunay triangulation and works well for point sets *P* of high density and uniform distribution. If the corresponding parameter alpha approaches 0, the alpha shape converges to *P*; whereas if alpha approaches ∞, the alpha shape converges to the convex hull of *P*. We set alpha to 90 voxels for all datasets. The obtained alpha shape is used to compute the volume, extract the surface and compute the surface area of the spheroid. Having obtained the surface of the spheroid, the relative position of each cell nucleus is determined by computing its distance to the surface.

#### Cell graphs

We derive two graph representations from the cell nuclei centroids representing the spatial distribution of cells within the cell aggregates. The proximity cell graph is given by *PCG*(*V, E*_*PCG*_) where *V* is the vertex set (i.e. the cell nuclei centroids) and *E*_*PCG*_ is the edge set of the graph. In the PCG, cells are neighbors if they are closer than a certain distance (*EdgeDistanceThreshold*). Thus, we obtained an edge (*u, w*) between two vertices *u* and *w* if the Euclidean distance between *u* and *w* is less than a predefined threshold of 40 voxels. The Delaunay cell graph is given by *DCG*(*V, E*_*DCG*_) where *V* is the vertex set and *E*_*DCG*_ is the edge set of the graph. The DCG graph is constructed based on a Delaunay triangulation to approximate which cells are in physical contact. An edge (*u, w*) was created between two vertices *u* and *w* if the corresponding points are connected by a line in the Delaunay triangulation and the Euclidean distance between *u* and *w* was less than a predefined threshold (*EdgeDistanceThreshold*) of 40 voxels. Consequently, the edge set *E*_*DCG*_ is a subset of *E*_*PCG*_ and the vertex set *V* for both graphs is identical. Edge weights in both graphs are equal to the Euclidean distance between the corresponding vertices. We extract the degree of each vertex as the number of neighbors. Further, the minimum, maximum, mean and standard deviation of the distance to neighbors for each vertex *v* is given by the weights of all edges incident to *v*.

#### Random cell position model

We compared the generated cell graphs to those of a mathematical random cell position (RCP) model. In this model, we made the following assumptions: (1) cells are distributed uniformly within a spheroid. (2) Cell nuclei are represented as non-overlapping spheres with positions drawn from a uniform random number distribution. The radius of the spheres was set to the median cell nucleus radius (~6 voxels) from all datasets. (3) Cell nuclei positions are restricted to the volume of the spheroid (i.e. the alpha shape) and are not allowed outside this volume. (4) The number of randomly generated cell nuclei is the same as the number of cell nuclei determined for the real spheroid. For each dataset, we performed ten Monte Carlo simulations of the RCP model and obtained the proximity and Delaunay cell graphs in the same way as for the real datasets. In other words, we generated an exact analogue of each cell aggregate.

### Implementation details

#### Implementation

The image analysis pipeline was developed in *Mathematica* version 10.2 (Wolfram Research, Inc.). A package containing the custom code of the image analysis pipeline, a user interface and two example datasets are available for download at http://www.physikalischebiologie.de/downloads. Computations were conducted on a workstation that comprises two six-core CPUs (X5650, Intel Corporation), 96 Gigabyte DDR3 memory, running Windows Server 2012 R2. Cell nuclei segmentation and post-processing took between five minutes and less than one hour per stack. The current implementation is parallelized at the initial cell nuclei segmentation step. For all subsequent steps, the complete image needs to fit into the main memory.

#### Three-dimensional reconstruction

Three-dimensional reconstructions were generated using the *Arivis* software (http://www.arivis.com).

## Additional Information

**How to cite this article**: Schmitz, A. *et al*. Multiscale image analysis reveals structural heterogeneity of the cell microenvironment in homotypic spheroids. *Sci. Rep.* **7**, 43693; doi: 10.1038/srep43693 (2017).

**Publisher's note:** Springer Nature remains neutral with regard to jurisdictional claims in published maps and institutional affiliations.

## Supplementary Material

Supplementary Information
