# Supplementary material for: Multiscale image analysis reveals structural heterogeneity of the cell microenvironment in homotypic spheroids
Source: Sci Rep. 2017 Mar 3;7:43693. doi: 10.1038/srep43693 (PMC5334646; doi:10.1038/srep43693)
Supplement: Supplementary Information [file srep43693-s1.pdf]

Supplementary Information

Multiscale image analysis reveals structural heterogeneity of the cell microenvironment in homotypic spheroids

Alexander Schmitz°, Sabine C. Fischer°*, Christian Mattheyer, Francesco Pampaloni and Ernst H.K. Stelzer

Physical Biology / Physikalische Biologie (IZN, FB 15)
Buchmann Institute for Molecular Life Sciences (BMLS)
Cluster of Excellence Frankfurt – Macromolecular Complexes (CEF – MC)
Goethe Universität – Frankfurt am Main (Campus Riedberg)
Max-von-Laue-Straße 15 – D-60348 Frankfurt am Main – Germany
°Authors contributed equally, *Corresponding author
*Correspondence to: sabine.fischer@physikalischebiologie.de

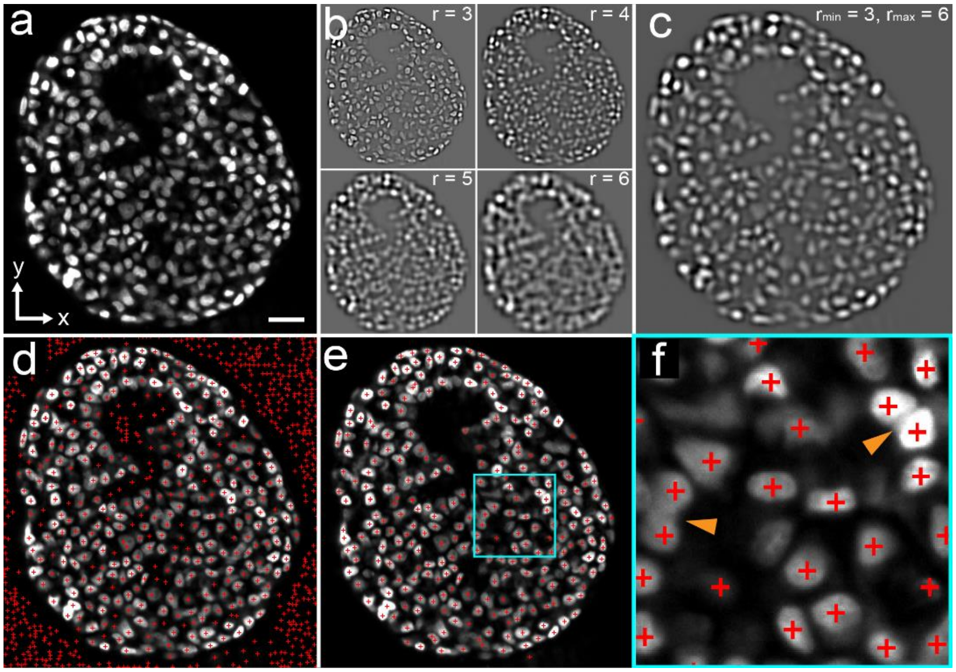

Supplementary Figure 1 Marker point detection with the Laplacian of Gaussian filter. (a) Sub-region of a single plane of the raw volume along X-Y. (b) Resulting images of the Laplacian of Gaussian (LoG) filter applied to the image in (a) for different values of the LoG filter range $\boldsymbol{r}$ in pixels. (c) The response images for different values of $\boldsymbol{r}$ are combined to provide a maximum response image. (d) Marker points detected by an extended local maxima search displayed as red crosses in the raw image. (e) Detections in the background region are discarded by multiplying the detected maxima image with the initial segmentation result. (f) Magnification of the indicated region (cyan box) in panel e. The marker point detection method accurately detects cell nuclei. For apparently touching cell nuclei (orange arrowheads), unique marker points are identified that define starting points for the subsequent watershed algorithm. Please note that for the purpose of this illustration, the marker point detection was applied in two dimensions. In the pipeline it is applied in three dimensions Scale bar: 25 µm.

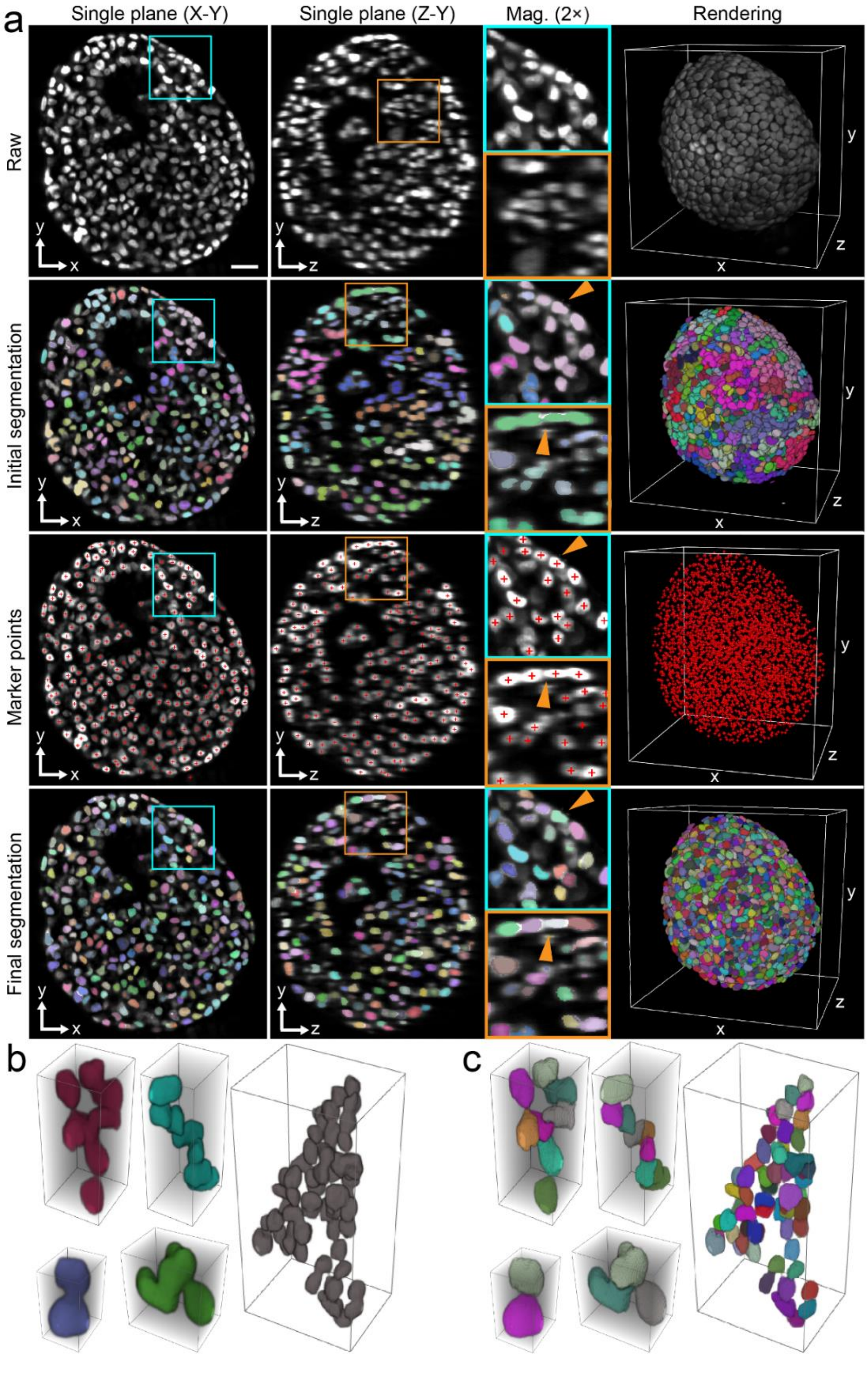

Supplementary Figure 2 Overview and evaluation of the steps of the cell nuclei segmentation. The individual steps of the cell nuclei segmentation are shown for dataset S9. Shown are single planes along X-Y (first column), Z-Y (second column) at about the center of the spheroid, two-fold magnifications (Mag. 2×) of regions indicated by cyan and orange boxes (third column) and three-dimensional renderings (fourth column). The obtained components after the initial segmentation are colored and overlaid on the raw image (second row). The range parameter $\boldsymbol{r}$ for the initial segmentation was set to 12 pixels. The initial segmentation identifies the foreground region in the image, but fails to separate apparently touching cell nuclei (orange arrowheads). Unique marker points (indicated by red crosses) are identified by the LoG filter (third row). Parameter values for $\boldsymbol{r_{min}}$ and $\boldsymbol{r_{max}}$ were set to three and six, respectively. In the final segmentation (fourth column), apparently touching cell nuclei are separated (orange arrowheads). Three-dimensional renderings of clusters of connected cell nuclei after connected component labelling of the initial segmentation (b) and after the final segmentation (c). Different colors represent individual components. Please note that to generate the images in the third row, the marker point detection was performed in two dimensions. In the image analysis pipeline, the marker detection is three-dimensional. Scale bar: 25 µm.

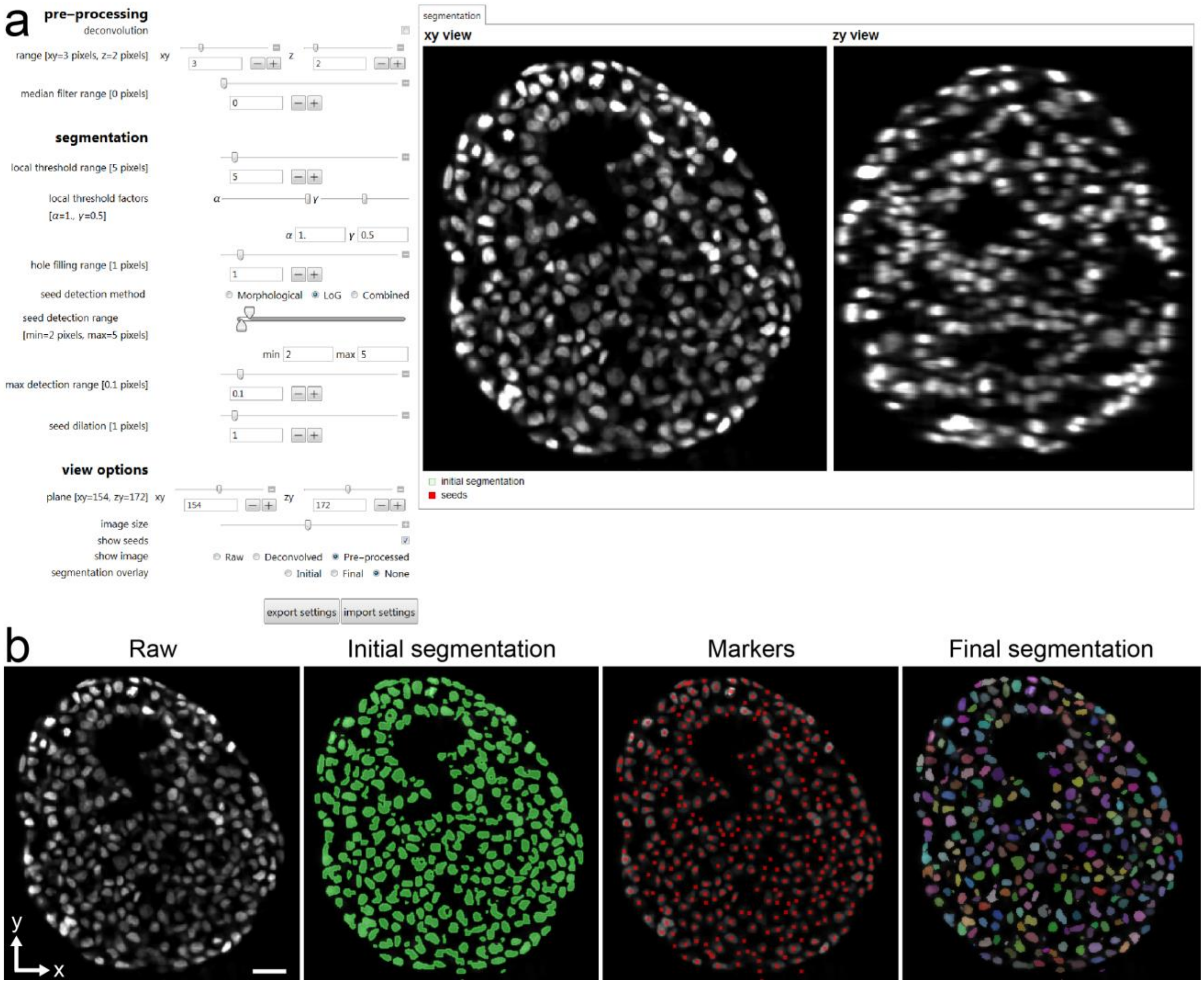

Supplementary Figure 3 User interface for cell nuclei segmentation. (a) Main user interface with control elements to adjust values of the segmentation parameters and two panels of the current image plane in X-Y and Z-Y. (b) Different modes of visualization overlaid on the raw image. The initial segmentation, the detected marker points and the final segmentation result after watershed. Note that the program will display the segmentation result for the current image plane and continuously update the displayed image. The chosen settings are then used for three-dimensional segmentation. The dataset shown is S9. Scale bar: 25 µm.

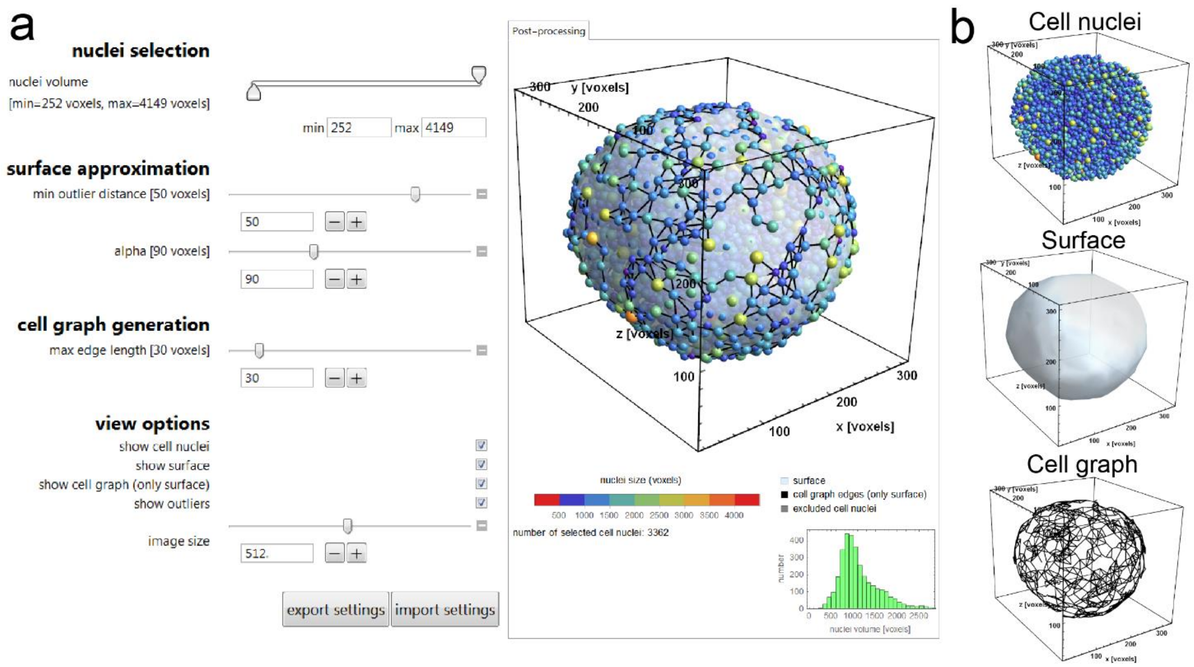

Supplementary Figure 4 User interface for post-processing. (a) Main user interface with control elements to adjust the values of the post-processing parameters. The resulting selection of cell nuclei as well as the surface of the alpha shape and the cell graph are continuously updated and shown on the right. (b) Different modes of visualization. The images depict cell nuclei rendered as spheres and colored according to the volume, the alpha shape surface and the cell graph for cell nuclei on the surface. The dataset shown is S9.

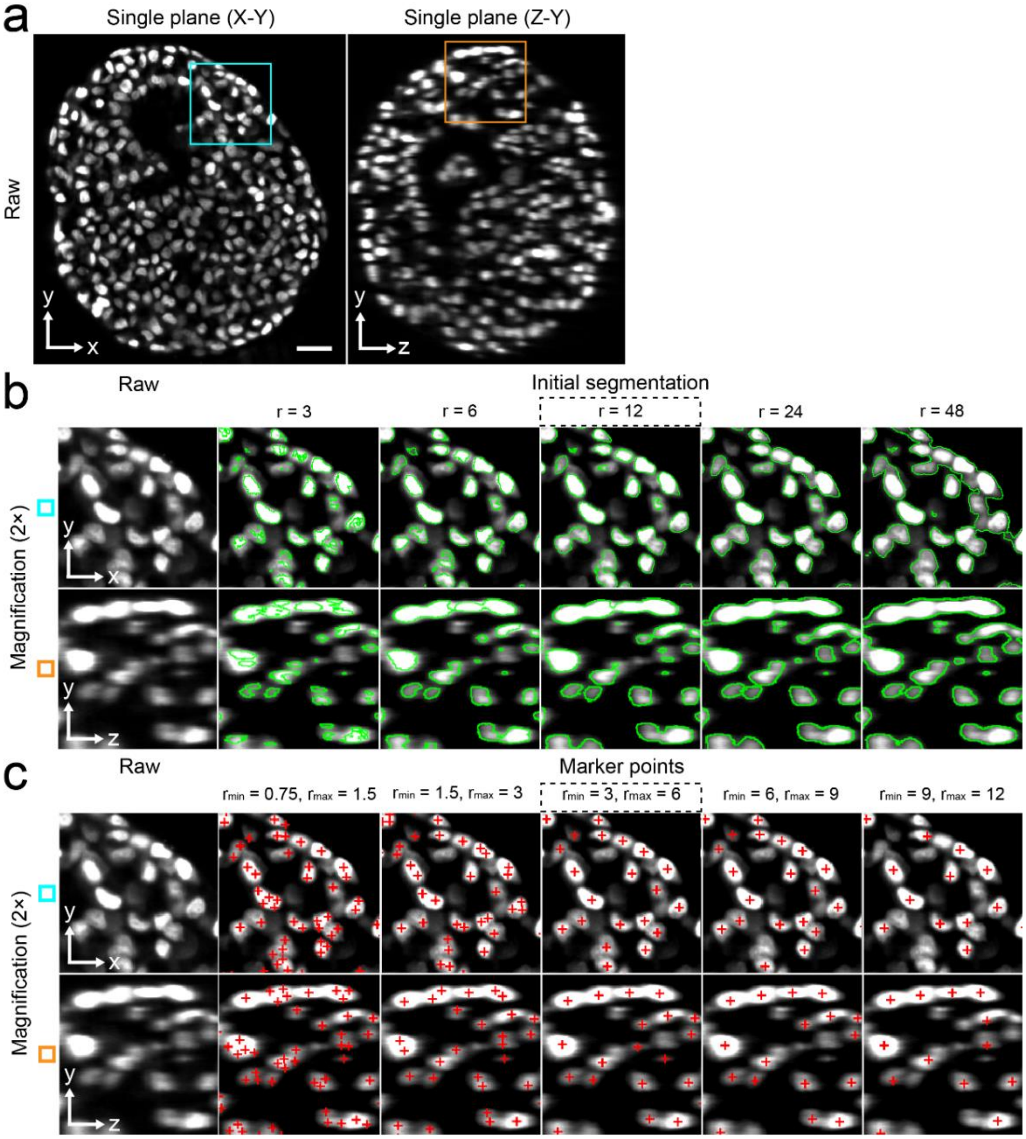

Supplementary Figure 5 Evaluation of parameter values for initial segmentation and marker point detection. (a) Representative single planes along X-Y and Z-Y of dataset S9 at the center of the spheroid. Scale bar: 25 µm. The results of initial segmentation (outlined in green) for different values of the range parameter $\boldsymbol{r}$ (b) and the results of marker point detection (depicted by red crosses) for different values of the parameters $\boldsymbol{r_{min}}$ and $\boldsymbol{r_{max}}$ (c) are shown in two-fold magnification for the regions indicated by cyan and orange boxes in panel a. The parameter values that were used in our study are indicated by a dashed box. Please note that the initial segmentation was applied as described in the Methods section, whereas for illustration purposes the marker point detection was applied in two dimensions. In the image analysis pipeline, the marker point detection is performed in three dimensions. A reasonable starting value for the range parameter of the initial segmentation is the average diameter of cell nuclei (about 12 pixels in the underlying datasets). However, the initial segmentation proved to be robust towards small deviations. Over-segmentation and under-segmentation occur for large deviations. For the marker point detection, reasonable values for the parameters for $\boldsymbol{r_{min}}$ and $\boldsymbol{r_{max}}$ are the minimal and maximal radius of cell nuclei in the dataset (three pixels for $\boldsymbol{r_{min}}$ and six pixels for $\boldsymbol{r_{max}}$). The marker point detection is more sensitive to the choice of the parameter values. For example, lower values (e.g. $\boldsymbol{r_{min}}$=1.5, $\boldsymbol{r_{max}} = \boldsymbol{3}$) lead to the detection of parts of cell nuclei, whereas for higher values (e.g. $\boldsymbol{r_{min}}$=9, $\boldsymbol{r_{max}} = \boldsymbol{12}$) clusters of cell nuclei are detected instead of individual cell nuclei.

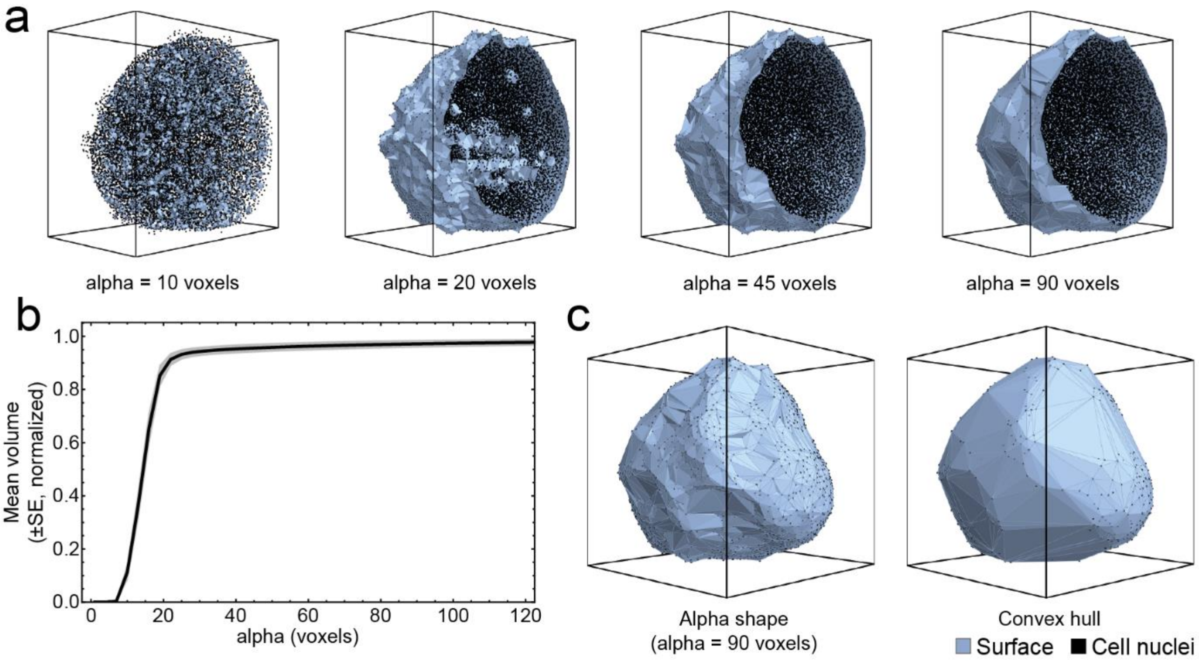

Supplementary Figure 6 Surface approximation using alpha shapes. (a) Alpha shape surface of dataset M3 for different values of the parameter alpha. Cell nuclei centroids are plotted as black points. (b) Mean volume of all datasets normalized to the volume of the convex hull as a function of alpha. The shaded region indicates the standard error of the mean (SE). (c) Alpha shape surface for alpha set to 90 voxels (left) and convex hull (right) for dataset M3.

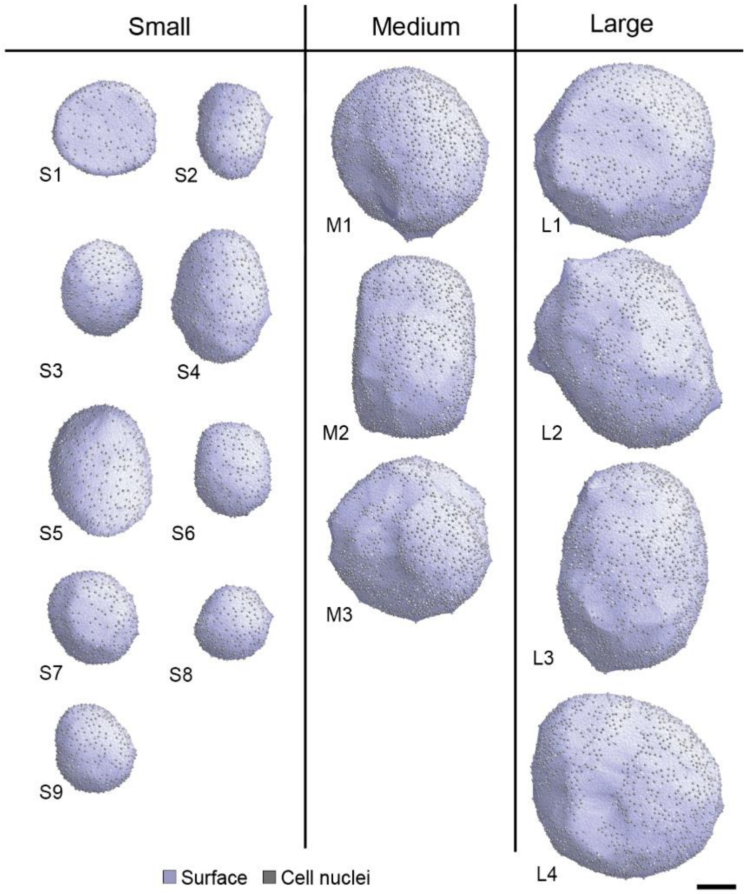

Supplementary Figure 7 Surface approximation for all datasets. For a complete list of datasets see Supplementary Table 4. Scale bar: 50 µm.

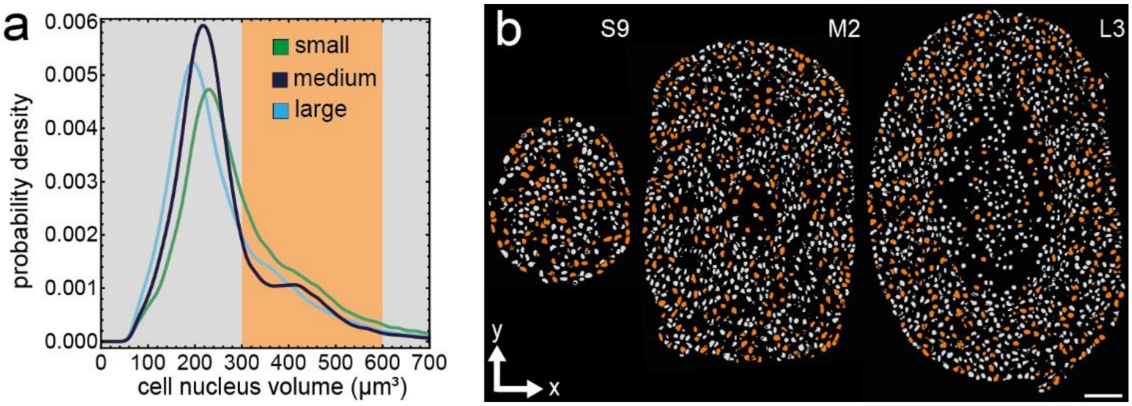

Supplementary Figure 8 Spatial localization of cell nuclei with a volume between 300 and 600 µm³. (a) Smoothed histogram of the cell nuclei volume distribution of small, medium and large spheroids. Cell nuclei with volumes in the region highlighted in orange (300 to 600 µm³) were colored in the segmentation images to analyze their spatial localization. (b) Single planes of datasets S9, M2 and L3 at the center of the spheroid. All cell nuclei with a volume of less than 300 µm³ or greater than 600 µm³ are depicted in gray. The cell nuclei with a volume between 300 µm³ and 600 µm³ are colored in orange. Scale bar: 50 µm.

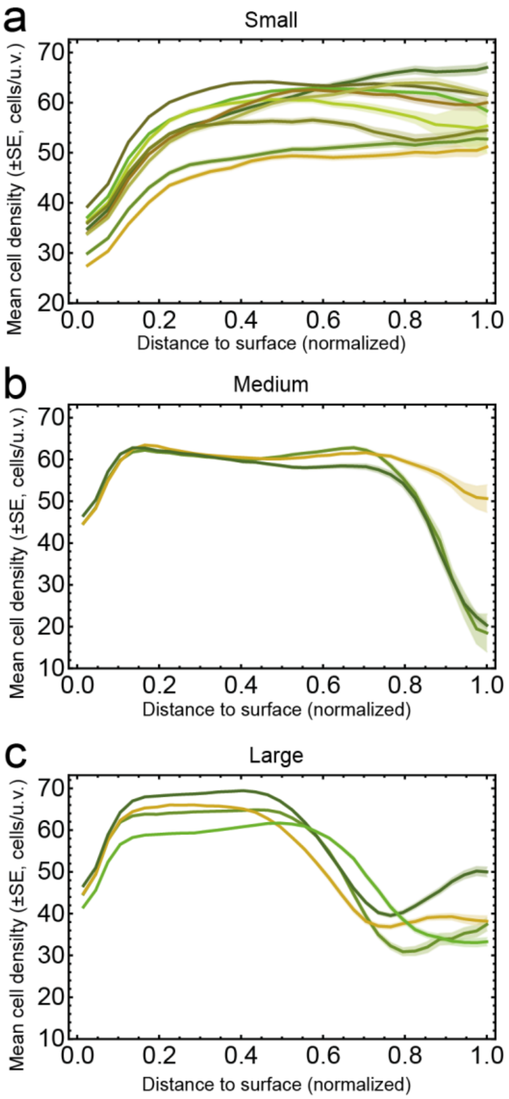

Supplementary Figure 9 Plots of the mean cell density versus the normalized distance to the surface for small (a), medium (b) and large (c) spheroids.

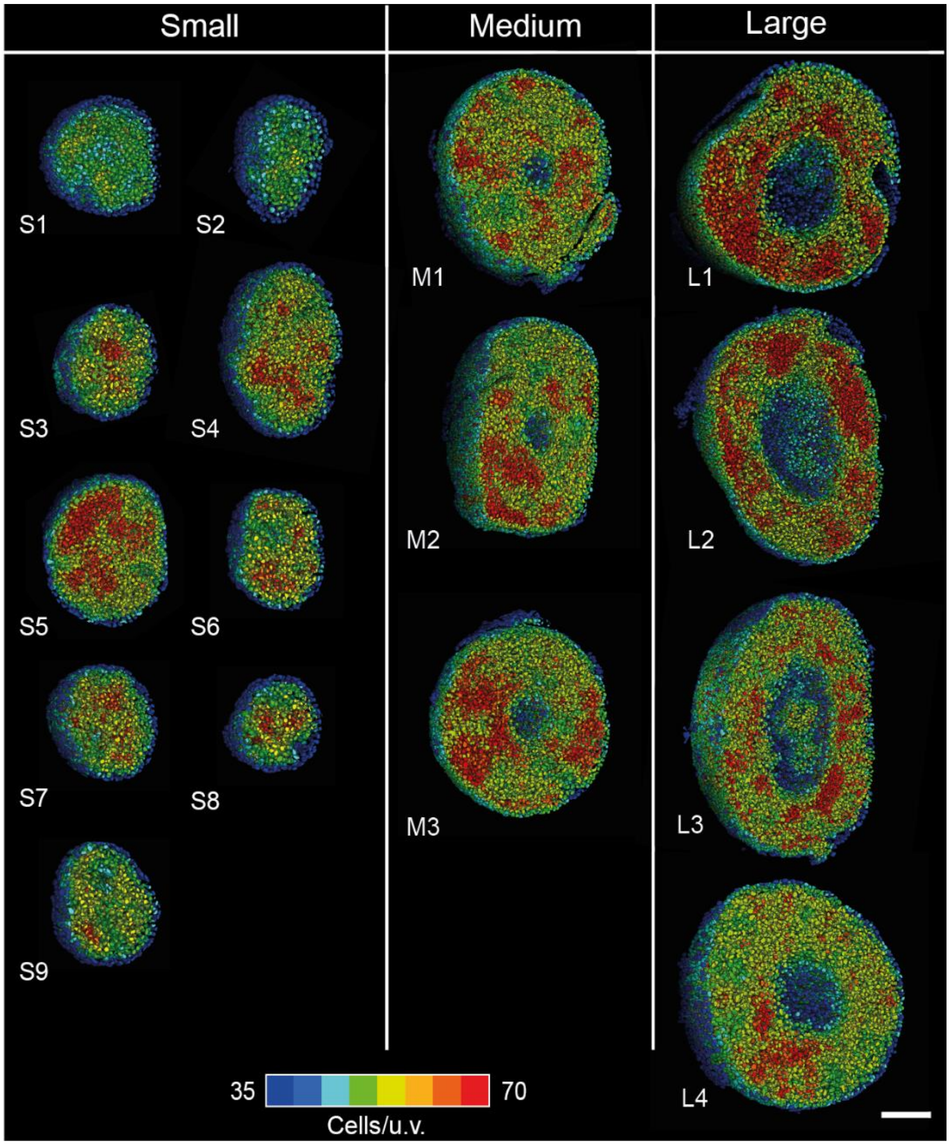

Supplementary Figure 10 Local cell density in all datasets. Three-dimensional rendering of segmented cell nuclei colored according to their corresponding cell density value for all small, medium and large spheroids, ranging from blue (35 cells/u.v.) to red (70 cells/u.v.). Renderings were clipped at the center of the spheroids. For a complete list of datasets see Supplementary Table 3. Scale bar: 50 µm.

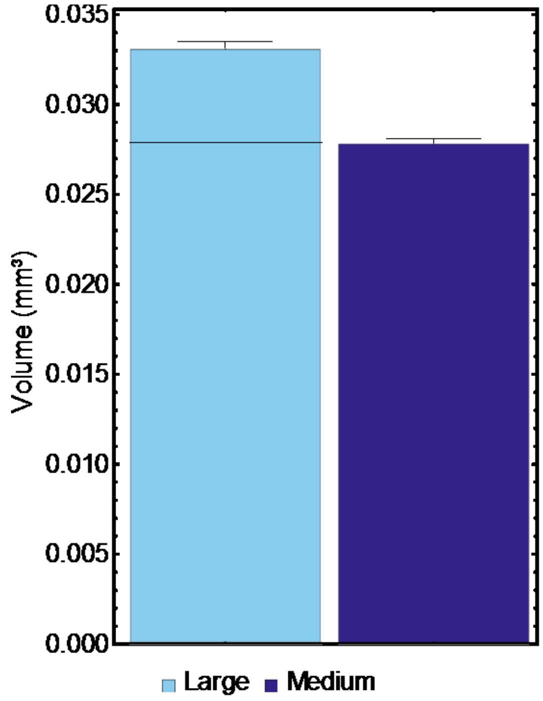

Supplementary Figure 11 Volume of outer region in medium and large spheroids.

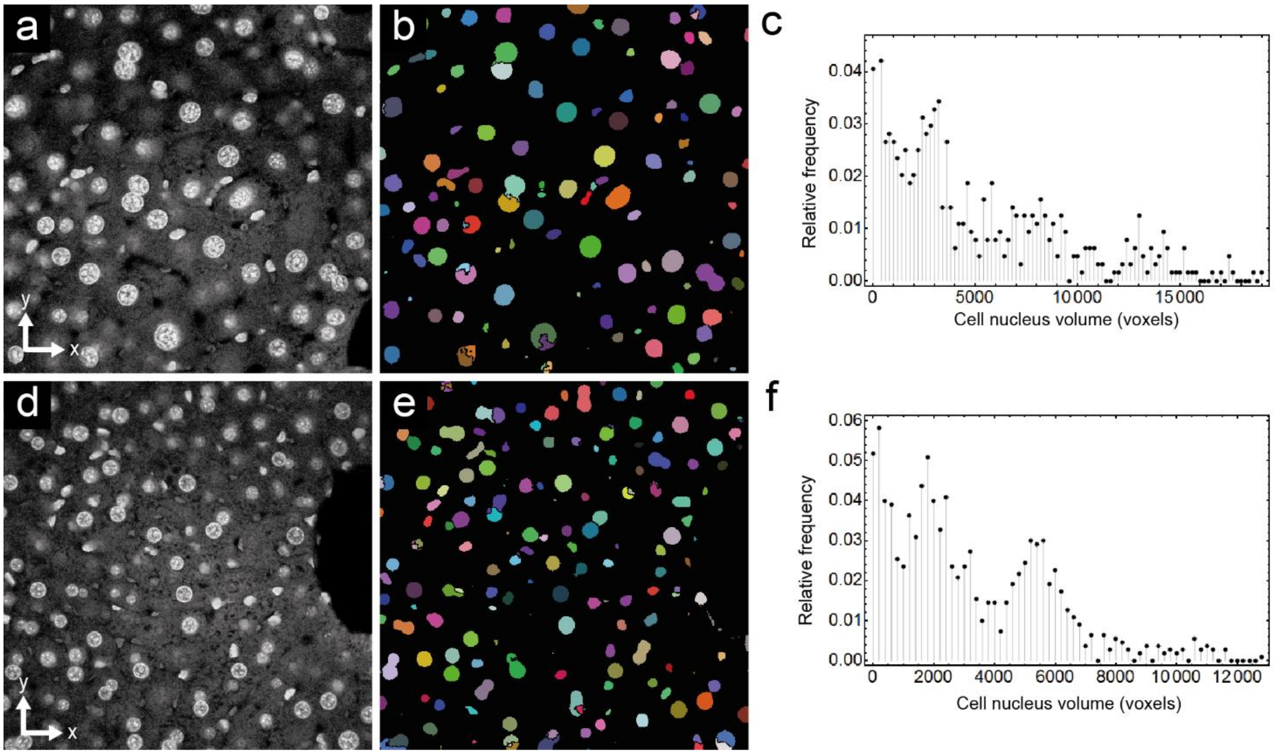

Supplementary Figure 12 Quality of cell nuclei segmentation in two test datasets from mouse liver imaged with a confocal microscope (Friebel *et al.*, *Bioinformatics*, 2015). (a) Single plane 38 along X-Y of test dataset #1 downloaded from (http://ms.izbi.uni-leipzig.de/index.php/software, accessed 11/10/2016). (b) The obtained components after cell nuclei segmentation are colored. (c) Plot of the relative frequency of cell nuclei volumes in the whole dataset #1. (d) Single plane 38 along X-Y of test dataset #2 downloaded from (http://ms.izbi.uni-leipzig.de/index.php/software, accessed 11/10/2016). (e) The obtained components after cell nuclei segmentation are colored. (f) Plot of the relative frequency of cell nuclei volumes in the whole dataset #2. For both data sets, one voxel corresponds to 0.02 μm³.

Supplementary Table 1 Evaluation of segmentation performance for different regions within a spheroid. The performance was measured against a manually determined ground truth for the regions I, II and III. The performance metrics recall, precision and F score are determined from the number of true positives, false negatives and false positives. Values range from 0 (worst performance) to 1 (optimal performance). GT, number of cell nuclei in the ground truth; SC, number of cell nuclei determined by the segmentation; TP, true positives; FN, false negatives; FP, false positives.

	**GT**	**SC**	**TP**	**FN**	**FP**	**recall**	**precision**	**F score**
**region I**	233	222	198	35	24	0.85	0.89	0.87
**region II**	269	262	230	39	32	0.86	0.88	0.87
**region III**	252	225	216	36	9	0.86	0.96	0.91

Supplementary Table 2 Parameter names and values used for cell nuclei segmentation, alpha shape and cell graphs. The same parameters values were used for all datasets. Please refer to the Methods section for a detailed description of the parameters.

Segmentation	
**Parameter**	**Value**
ImageZScalingFactor	3.5
ImageScalingFactor	0.5
NucleiSeedDetectionMinRadius	3
NucleiSeedDetectionMaxRadius	6
NucleiFilterRange	3
NucleiThresholdRange	12
NucleiMeanFactor	1
NucleiSeedDilation	2
NucleiBackgroundFactor	0.25
NucleiMinCount	250
NucleiMaxCount	42,500

Alpha shape / cell graphs	
**Parameter**	**Value**
Alpha	90
OutlierDistanceThreshold	20
EdgeDistanceThreshold	40

Supplementary Table 3 Overview of features extracted for the spheroid and individual cells.

	Feature Name	Description	Illustration
**Spheroid features**	*ProximityCellGraph (PCG)*	Proximity cell graph $PCG(V, E_{PCG})$ with vertices V and edges $\mathrm{E_{PCG}}$ describes the whole spheroid. Vertices V have positions $\{x, y, z\}$ and represent cell nuclei centroids. An edge $(u, w) \in E_{PCG}$ exists if the Euclidean distance between the two vertices u and w is less than a predefined threshold. The edge weight is determined by the Euclidean distance between the two vertices u and w	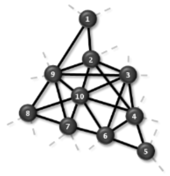
	DelaunayCellGraph (DCG)	Delaunay cell graph $DCG(V, E_{DCG})$ with vertices V and edges $\mathrm{E_{DCG}}$ describes the whole spheroid. Vertices V have positions $\{x, y, z\}$ and represent cell nuclei centroids. An edge $(u, w) \in E_{DCG}$ exists if it is part of the Delaunay triangulation of V and the Euclidean distance between the two vertices u and w is less than a predefined threshold. The edge weight is determined by the Euclidean distance between the two vertices u and w.	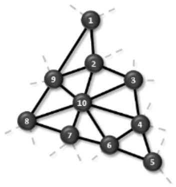
	Surface	Surface of the spheroid (light blue) constructed from all cell nuclei centroids P using the alpha shapes approach. Points in P located on the surface are shown in gray.	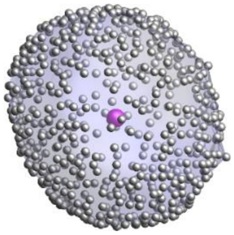
	Centroid	Geometric center $\{x, y, z\}$ of the spheroid (purple) is the arithmetic mean of all cell nuclei centroids.	
	MinDistanceSurface	Euclidean distance of *Centroid* to its closest point on *Surface*.	
	Volume	Total number of voxels of the volume enclosed by and including *Surface*.	
	SurfaceArea	The number of voxels of *Surface*.	

	Feature Name	Description	Illustration
**Cell nuclei features (segmentation, surface)**	*Label*	Unique integer that identifies a cell nucleus.	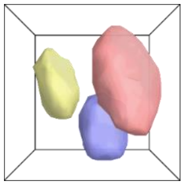
	Mask	A binary three-dimensional array, in which 1s refer to the voxels that are part of a cell nucleus.	
	BoundingBox	Axes-oriented minimal box $\{\{x_{min}, y_{min}, z_{max}\}, \{x_{max}, y_{max}, z_{max}\}\}$ that contains a cell nucleus.	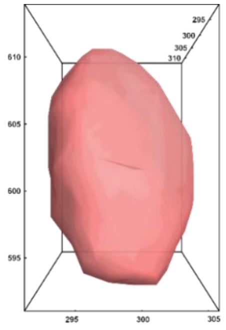
	Count	Total number of voxels of a cell nucleus.	
	PerimeterCount	Total number of voxels on the surface of a cell nucleus.	
	MinCentroidDistance, MaxCentroidDistance, MeanCentroidDistance	Minimum, maximum and average distance of all voxels to the geometric center of a cell nucleus.	

	Centroid	Geometric center $\{x, y, z\}$ of a cell nucleus (purple sphere).	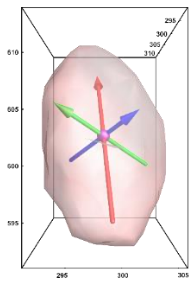
	MainAxes	Main axes of orientation $\{\vec{p}_1, \vec{p}_2, \vec{p}_3\}$ of a cell nucleus measured by principal components analysis. In particular, $\vec{p}_1$ (red) is the vector along the first, $\vec{p}_2$ (green) along the second and $\vec{p}_3$ (blue) along the third principal component, respectively.	
	Extension	Extension $\{e_1, e_2, e_3\}$ of a cell nucleus along its three main axes of orientation $\{\vec{p}_1, \vec{p}_2, \vec{p}_3\}$ as the number of voxels, where e_1 is the extension along $\vec{p}_1$, e_2 along $\vec{p}_2$ and e_3 along $\vec{p}_3$, respectively.	
	TotalIntensity, *MeanIntensity*, *StandardDeviationIntensity*, *MinIntensity*, *MaxIntensity*	Total, average, standard deviation, minimum and maximum value of the intensity distribution of a cell nucleus measured in the raw volume. Intensity features are normalized to the interval $[0, 1]$.	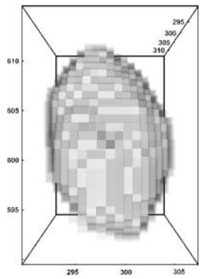
	IntensityCentroid	Intensity weighted geometric center $\{x, y, z\}$ of a cell nucleus.	
	SurfaceDistance, SurfaceNearest	Minimal distance (black arrow) of the cell nucleus centroid (magenta sphere) to the aggregate surface (*Surface*, light blue) in number of voxels, and the corresponding surface point $\{x, y, z\}$.	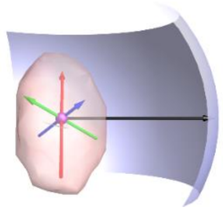
	SurfaceOrientation	Angle in degree between major axis vector $\vec{p}_1$ (red) of the cell nucleus and the aggregate surface (*Surface*, light blue). An angle of 0° indicates that $\vec{p}_1$ is parallel to *Surface*, whereas for an angle of 90°, $\vec{p}_1$ is orthogonal *Surface*.	

	**Feature Name**	**Description**	**Illustration**
**Cell neighborhood features (cell graphs)**	*NeighborCount*, *MinNeighborDistance*, *MaxNeighborDistance*, *MeanNeighborDistance*, *StandardDeviationNeighborDistance*	Number of vertices adjacent to a vertex v (e.g. vertex 10 in the illustration has seven neighbors). Minimum, maximum, mean and standard deviation of the edge weight of all edges incident to a vertex v. These features are derived from both proximity cell graph and Delaunay cell graph.	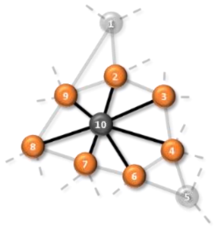

Supplementary Table 4 Quantitative measures for all datasets. Based on automated cluster analysis (partitioning around medoids with squared Euclidean distance) of spheroid volume and cell number, the datasets were separated into three groups of small (S1-S9), medium-sized (M1-M3) and large (L1-L4) spheroids.

**Dataset**	**Group**	**Number of seeded cells**	**Volume (µm³)**	**Number of cells**	**Median cell nucleus volume (µm³)**	**Median absolute deviation of cell nucleus volume (µm³)**
**S1**	Small	1,000	$5{,}81\times10^{6}$	4,155	333	77
**S2**	Small	1,000	$4{,}15\times10^{6}$	2,883	341	78
**S3**	Small	1,000	$4{,}68\times10^{6}$	3,981	263	57
**S4**	Small	2,000	$1{,}06\times10^{7}$	8,975	245	57
**S5**	Small	2,000	$1{,}18\times10^{7}$	10,311	249	55
**S6**	Small	500	$4{,}58\times10^{6}$	3,938	268	71
**S7**	Small	500	$5{,}96\times10^{6}$	4,866	260	66
**S8**	Small	500	$2{,}71\times10^{6}$	2,326	261	77
**S9**	Small	500	$3{,}98\times10^{6}$	3,359	280	66
**M1**	Medium	5,000	2.90×10^{7}	25,775	234	51
**M2**	Medium	5,000	2.88×10^{7}	25,908	235	48
**M3**	Medium	5,000	2.82×10^{7}	25,064	232	53
**L1**	Large	10,000	3.80×10^{7}	32,898	211	50
**L2**	Large	10,000	4.10×10^{7}	35,097	211	53
**L3**	Large	10,000	4.21×10^{7}	38,752	209	54
**L4**	Large	5,000	3.87×10^{7}	32,222	279	62
